# Shall the London University Institute a Degree in Dentistry?

**Published:** 1904-03-15

**Authors:** 


### SHALL THE LONDON UNIVERSITY INSTITUTE A DEGREE IN DENTISTRY?

A meeting of considerable importance to the dental profession was held at Examination Hall, Royal College of Surgeons, London, on the 23d ultimo, to discuss and to vote upon this question. Mr. Walter Harrison, President of the British Dental Association, occupied the chair.

Between two hundred and three hundred members were present, and the profession was represented from all parts of the country.

The Chairman opened the proceedings by stating that at the annual meeting of the Association held at Brighton, in June last, a resolution was passed expressing a desire that the representative board should consider the advisability of presenting a memorial to the Senate of the University of London on the question of granting degrees, and take action thereon. The resolution on that occasion was that the board had no power to do this. The matter came before the representative board in November, when the board passed a resolution referring the subject to an extraordinary meeting of members to be held on the 23d of January. The Chairman then briefly announced to the meeting what his method of procedure would be, and what resolutions and amendments would be brought before them to consider. He then called upon Mr. J. H. Badcock to move the resolution which stood in his name, viz:

"That a memorial be presented to the Senate of the University of London praying for the institution, in the interests of dental science, the public, and the dental profession, of a Degree of Dental Surgery in the Faculty of Medicine; but expressing the further opinion that it would be undesirable to make the possession of the M.B. B.S. degrees a necessary qualification for such a degree."

Mr. F. J. Colyer rose to suggest that, as the meeting was a large one, and many had come from a distance to speak, a time limit should be put upon the speeches.

The Chairman did not propose to place any limitation on the speakers in the early part of the discussion, though he thought it might be necessary later.

Mr. J. H. Badcock, who was received with loud cheers, said: "It was common knowledge that for some time past the University of London had been undergoing reconstruction; it had become a teaching body, and lately it had been considering the advisability of establishing a Degree of

Dentistry. The speaker thought it would be well for the profession to form its own opinion on the subject and express it. He believed a degree in dental surgery instituted by London University would be a very desirable thing, that it would have an educational value, raise the status of the profession, and be an example to other universities. At the present time the curricula of the four examining bodies varied somewhat in standard, but not in scope. There were two sides to the profession, the scientific and research side, and the side which was engaged in actual daily practice. As a qualification for actual practice he did not think they could have anything better than the L.D.S., but he might call the 'Intelligence Department' of the profession, for what he did not think it was adequate. He thought this view was borne out by the poverty of English dental literature, and the scarcity of young men engaged in research work. If this side of the profession were neglected England must inevitably fall behind other nations. The L.D.S., excellent though it was as a guarantee of efficiency for every day practice, was not sufficient for the man of science whose studies must be wider and deeper. To the answer 'take the conjoint,' he would reply that taking the membership consumed time and energy in a wrong direction for the object they had in view; it would be better to devote the time more strictly to the mission in life or which the scientific investigator had set himself, and a special honor degree would do this. He would call the L.D.S. the 'Pass Diploma,' entitling to register and to practice, and the honors degree would be the incentive to the attainment of the maximum of knowledge, and to ever push forward its bounds by research work. As to the effect it would have upon the status of the profession, he thought it would raise it. There were some who seemed to think that dental surgery had no status apart from the medical profession; that it enjoyed a reflected effulgence. He believed that it was something more than the 'poor relation' of general surgery, and *had* a status of its own. A very important point in connection with the subject was the undesirability, in the present state of supply and demand, of further raising the curricula for license to practice. Already there were an insufficient number of qualified dentists for the needs of the population, and men were not coming into the profession fast enough. If the demand were not met, quackery would flourish, for substitution was the only effective method of stamping it out. He then dealt with the 'one portal' question, and felt upon that point they were quite safe, because no licensing body had power to grant more than the L.D.S. Finally, whether they liked it or not, other universities would grant a dental degree—Birmingham had already done so—was London going to lag behind?" (Prolonged applause.)

Mr. Leonard Matheson, whose rising provoked a fresh outburst of cheering, seconded the resolution. He approached the question with very mixed feelings—not that he wavered in his convictions, or hesitated as to the right course, but that it was painful to him to be in antagonism with the friends of his life. Mr. Badcock had said if a degree were established by London University it need not, and probably would not, be a registrable degree. But suppose for a moment that that degree were somehow made registrable, and that it was not an "L.D.S." Would it compete with the L.D.S.? He (the speaker) said it could not, for it would only be taken by the few. For the "one portal" system he had a tender regard—it had such a pleasant, simple sound. But they must remember they had not got it. In addition to the four registrable diplomas—London, Edinburgh, Glasgow and Dublin—there were the medical diplomas and certain foreign diplomas which gave title to practice. It was said again that a university degree would injure the honor and prestige of the L.D.S. Take his own case, for he held no other diploma or degree than the L.D.S. He heard some one saying, "My dear Matheson, think of it! Your reputation will be gone. You will never be able to lift up your head again." Away with such unworthy thoughts. Was he to be afraid for his pocket? Was he to lose the esteem of his patients? Was he to be cold-shouldered by his professional brethren? But, it might be said, "Have a little common sense. Imagine some little country town, what chance would the L.D.S., have against a University degree? How would he like it then?" He answered, if he were poor in attainment, poor in *savoir faire*, poor in spirit, poor in every thing that made a professional man, he should be very much annoyed, he should be very bitter, he should be very angry. But if he knew his work, and could do good work, he would not be concerned, for he would have the esteem of his patients. It was what a man is and does that counted, and what he was called:

"A man's a man for a' that."

Were they a trade union or a profession? Did they, or did they not, subscribe to the doctrine of one dead level for all? What about their relation with medicine? If they granted that the L.D.S. united them to the medical profession then he contended a degree in dentistry would still more unite them, for it would give them wider, higher, and better knowledge, and medical men would with ever-growing confidence turn to them and look to them as specialists and consultants. The new degree would give them a wider base and a higher apex. (Loud cheers.)

The Chairman, having ruled the first part of Mr. Rees Price's amendment out of order, called upon him to move the second part, at the same time ruling that it was tantamount to "the previous question," and, if carried, would end the discussion.

Mr. Rees Price, in the circumstances, asked leave to withdraw the amendment.

Mr. Balding, being called upon, asked leave to withdraw his amendment in favor of Mr. Hepburn's, which, he said, covered the same ground but went rather further.

Mr. David Hepburn moved the following amendment:

"That the creation of degrees in dentistry is undesirable, and that the opinion of this meeting, especially summoned to consider the expediency of a Degree in Dentistry, be communicated to the principals of the universities of the United Kingdom."

He said he could not lay claim to the eloquence which had characterized the two opening speeches, but in a few plain words he would state his position. He first dealt with the circumstances under which the circular to which reference had been made was issued. It was not for the purpose of organizing opposition, but he would hardly have felt justified in pressing his personal views without endeavoring to ascertain to what extent they had the support of the profession. The subject seemed to divide itself under two heads: 1, their present position; 2, the dangers and difficulties of the proposed change. He need not refer back in detail to the time in the history of the profession when a great struggle took place which resulted in the institution of the L.D.S. It was by that license that they became a profession. By that they became an integral part of the medical profession. In the public mind there was now no confusion or excuse; they were now beginning to know and to realize the credentials entitling a man to practice dentistry; they were now recognizing that the L.D.S. was the only guarantee for skillful and professional treatment. Were they going to unsettle the public mind and once more create the confusion upon which charlatanism thrived? (Cheers.) Though the license was granted by four colleges, yet it was a one portal system. He felt very strongly that the proposed University Degree would tend to separate them from the medical profession. He viewed with disfavor any proposal that would have that tendency, and looked for that social and educational advancement through channels which would link them more closely to, not separate them from, the medical profession. Who was working for the change? Was it the teachers? Was it the authors? Was it the research workers? Was it the public? The multiplication of degrees by the growing number of universities in so small a specialty as dentistry would result in a vast confusion which would be nothing else than grotesque. (Cheers.) It would be a grand opportunity for the unscrupulous to take to themselves pseudo titles! (Cheers.) Look at other countries. He would ask them, did they wish to see here what they saw there? Finally, many a hard-working professional man would view a new degree as an insult to their excellent diploma. (Loud cheers.)

Mr. Montagu Hopson seconded the amendment. If they made a request to the London University, they thereby invited other universities to grant similar degrees. Demand created supply, and supply reacted on demand. Competition would eventually result in lowering standards, and in lowering examination fees; they would then have a multiplicity of degrees in dentistry varying in educational value, and all tending to impair the value of the L.D.S. Some one had wittily said that the B.D.S. (Bachelor of Dental Surgery), would stand for Big Dental Surgeon, and the L.D.S., for Little Dental Surgeon—(laughter)—and undoubtedly in little country towns that would be the effect. It was argued that the new degree would not be registrable. But could it for a moment be expected that an important body like a university would consent to remain long in that invidious position? Would it stultify itself by conferring a so-called honor degree which carried with it no right to practice, while the pass diploma gave that right? Dentistry was and he hoped it would ever remain a special department of surgery. With what grace did they seek permission to use that building, that very theater, for the purpose of cutting themselves adrift from the medical profession. (Cheers.)

The Chairman now said that it was now 4 o'clock, there were many more speakers, and he must limit them to ten minutes each. (Shouts, "five!") Very well, gentlemen, five."

Mr. G. G. Campion then addressed the meeting. He had come with much to say, but felt himself embarrassed by the time limit. It was hardly possible, he said, to deal with a single point in five minutes. He wished to approach the question from the ultilitarian side. They knew the Dean of the Royal Dental Hospital was continually urging the student to take "the conjoint." But did the conjoint make a man a better dentist? The whole object of the dental degree—the B.D.S., was to make a man a "Better Dentist Still" (laughter), and a man who was "Master in Dental Surgery" he supposed would be a Much Better Dentist Still. (laughter).

Dr. Baker said Dublin University had not been petitioned by any members of the dental profession, but he had been approached by the university to draw up a curriculum. In granting this degree Trinity College proposed to take them on an equal standing with law, divinity and engineering. (Cheers). He then sketched in some detail the curriculum he would recommend.

Mr. Arthur Underwood emphasized the point that a university degree would inevitably lead to their separation from medicine. Innumerable degrees would collect dentistry in one place, and surgery and medicine would be in another. They must not think of to-day, but of twenty years hence.

Mr. Morton Smale spoke with unwonted animation, and vigorously attacked the proposed multiplication of degrees. He did not object to university recognition a bit, but let the degree be the L.D.S. When he stood behind a man like Mr. Brunton who held no diploma of any kind he asked would fifty degrees make a grander man of him than he was? (Cheers.)

Mr. Brunton said he considered this question not entirely from the dental stand-point, but from the national stand-point. They were not sufficiently numerous to keep abreast of the needs of the nation, of the navy, of the army, and of the schools. The point was, which was the way to make the best dentist? He thought the L.D.S. made the best dentist. Provided it remained compulsory to take the L.D.S., in order to be able to practice, and the university degree was only additional, he would not object to it, and he had come to the conclusion to vote for Mr. Badcock's resolution.

Messrs. Constant, Norman Bennett, Dennant, J. F. Colyer, W. Rushton, Baldwin, Spokes, Patterson, Robbins, Mountford, Oliver, and B. Cunningham also spoke.

Mr. Badcock having replied a vote was taken.

For the Amendment..........................175
Against.............................................. 87

The amendment was then put as an substantive motion, and declared carried by a show of hands.

The proceedings were throughout of an animated character, and vociferous cheering punctuated the points of the speeches.

A vote of thanks, proposed by Mr. Hepburn, and seconded by Mr. Badcock, to Mr. Harrison for his able conduct in the chair, brought the proceedings to a close.

